# *Bacillus licheniformis* strain POT1 mediated polyphenol biosynthetic pathways genes activation and systemic resistance in potato plants against Alfalfa mosaic virus

**DOI:** 10.1038/s41598-020-72676-2

**Published:** 2020-09-30

**Authors:** Ahmed Abdelkhalek, Abdulaziz A. Al-Askar, Said I. Behiry

**Affiliations:** 1 Plant Protection and Biomolecular Diagnosis Department, ALCRI, City of Scientific Research and Technological Applications, New Borg El Arab City, Alexandria Egypt; 2 Faculty of Science, Botany and Microbiology Department, King Saud University, Riyadh, Saudi Arabia; 3 Agricultural Botany Department, Faculty of Agriculture (Saba Basha), Alexandria University, Alexandria, 21531 Egypt

**Keywords:** Applied microbiology, Plant molecular biology, Biotic, Effectors in plant pathology, Microbe, Transcription

## Abstract

Alfalfa mosaic virus (AMV) is a worldwide distributed virus that has a very wide host range and causes significant crop losses of many economically important crops, including potato (*Solanum tuberosum* L.). In this study, the antiviral activity of *Bacillus licheniformis* strain POT1 against AMV on potato plants was evaluated. The dual foliar application of culture filtrate (CF), 24 h before and after AMV-inoculation, was the most effective treatment that showed 86.79% reduction of the viral accumulation level and improvement of different growth parameters. Moreover, HPLC analysis showed that a 20 polyphenolic compound was accumulated with a total amount of 7,218.86 and 1606.49 mg/kg in POT1-treated and non-treated plants, respectively. Additionally, the transcriptional analysis of thirteen genes controlling the phenylpropanoid, chlorogenic acid and flavonoid biosynthetic pathways revealed that most of the studied genes were induced after POT1 treatments. The stronger expression level of *F3H*, the key enzyme in flavonoid biosynthesis in plants, (588.133-fold) and *AN2*, anthocyanin 2 transcription factor, (97.005-fold) suggested that the accumulation flavonoid, especially anthocyanin, might play significant roles in plant defense against viral infection. Gas chromatography-mass spectrometry (GC-MS) analysis showed that pyrrolo[1,2-a]pyrazine-1,4-dione is the major compound in CF ethyl acetate extract, that is suggesting it acts as elicitor molecules for induction of systemic acquired resistance in potato plants. To our knowledge, this is the first study of biological control of AMV mediated by PGPR in potato plants.

## Introduction

Potato (*Solanum tuberosum* L.) is the third-largest economic food crop in the world, including Egypt, after rice and wheat^1^. Under field conditions, potatoes suffer from many viral diseases infections. Among such viruses causing great economic losses and considered the major limiting factors for potato production is alfalfa mosaic virus (AMV; genus *Alfamovirus*, family *Bromoviridae*)^2^. It is a worldwide-distributing virus, infecting 698 species of 167 genera in 71 families, including Solanaceae and Leguminosae^2–4^. It is transmitted by sap inoculation and by numerous aphid species in a non-persistent manner^3^. Most potato cultivars are susceptible to AMV infection and induce diverse symptoms, including yellow blotching and bright mottling of potato leaves resulted in calico symptoms^2, 5^. AMV management is difficult and depends mainly on selecting resistant plant cultivars and/or intensive insecticide and pesticide treatments that are often used to control its vector spreading^6^. The application of plant growth-promoting rhizobacteria (PGPR), as bio-control agents, is a promising safe approach in crop protection against different pathogens^7^, including viruses^6^. Different *Bacillus* spp. have been reported to induce antiviral responses against tomato spot wilt virus (TSWV) and potato virus Y (PVY) in tomato plants^8^ and against cucumber mosaic virus (CMV) in tobacco^9^, tomato^10^, *Arabidopsis*^11^. Under field conditions, the application of powder and liquid formulations of three PGPRs strains (*Bacillus Licheniformis*, *Bacillus* spp., *Pseudomonas aeruginosa*) reduced the severity of sunflower necrosis virus (SNV) up to 51.4% in sunflower plants^12^.

Generally, PGPRs acting as elicitor activate the systemic resistance through induced systemic resistance (ISR) either by salicylic acid (SA) signaling pathway and/or jasmonic acid (JA) pathways^13–17^. Furthermore, PGPRs can produce and induce a wide diversity of useful bioactive metabolites^15^. Potato polyphenolic compounds are one of these secondary metabolites that played vital roles in plant growth development and protection against various biotic and abiotic stresses^18,19^. A recent area of research is of particular interest is their antiviral activity. Through intercalating of its B ring with viral nucleic acid bases or viral capsid proteins, flavonoids can inhibit viral polymerases enzymes^18,20,21^. Through transporting to the site of infection and incorporating into the cell walls of necrotic and adjacent cells, flavonoid compounds can induce a hypersensitivity reaction, the first defense mechanism of infected plants, and programmed cell death^22–25^.

Potatoes are good sources of chlorogenic acid and flavonoids, which constitute the majority of polyphenolic compounds^26^. Induction and expression of such genes were correlated with polyphenol content under both normal and stress conditions^27^. The present study evaluated the efficacy of *Bacillus licheniformis* strain POT1 to induce systemic resistance in potato against AMV infection and its effects on plant growth development and transcriptional levels of phenylpropanoid, chlorogenic acid and flavonoids biosynthetic pathways genes. Furthermore, identification of the metabolites in potato plant extract and the bioactive constituents of POT1 crude filtrate were performed using High-performance liquid chromatography (HPLC) and Gas chromatography-mass spectrometry (GC-MS) analysis.

## Results

### Bacterial isolation phenotypic identification and molecular characterization

By using the half-leaf method^28^, the most potent bacterial isolate exhibiting antiviral activity was selected and subject for morphological, physiological, biochemical characteristics (Table 1) and molecular identification. Based on the bacterial phenotypic characteristics and nucleotide sequencing analysis of the amplified 16S rRNA gene, the selected bacterial isolate was identified as *Bacillus licheniformis* strain POT1, and the annotated sequence was submitted to GenBank database under the accession number MT077309. The NCBI-BLAST alignment analysis indicated that POT1 isolate was closely related to other *B. licheniformis* isolates especially that isolated from India (Acc# MG428771) and (Acc# MH733009). The phylogenetic tree analysis indicated a high identity of *B. licheniformis* isolates from different parts of the world with 99% and presented as a monophyletic group of these isolates (data not shown).

**Table 1:** Morphological, physiological and biochemical characteristics of *Bacillus licheniformis* POT1 isolate.

Characteristics test	Result*
Shape(rods in chain)	+
Gram staining	+
Motility	+
Anaerobic growth	+
Spore formation	+
Growth at 30–55 °C	+
Nitrate Reduction	+
Citrate Utilization	+
H2s production	−
Catalase production	+
Urease production	+
Growth in 7% NaCl	+
Growth on Skimmed milk	+
Indole production	−
Gelatin decomposition	+
Melibiose	−
Dulcitol	−
Raffinose	−
L-alanine	−
D-galacturonic acid	−
Glucose	+
Lactose	−
Maltose	a
Glycerol	a
Fructose	a
Sucrose	a
Manitol	a
Galactose	a

* + , ≥ 81% positive reactions; −, ≤ 19% negative reactions; a, acid production.

### Effect on symptom development, AMV accumulation level and growth parameters

**Figure 1:**
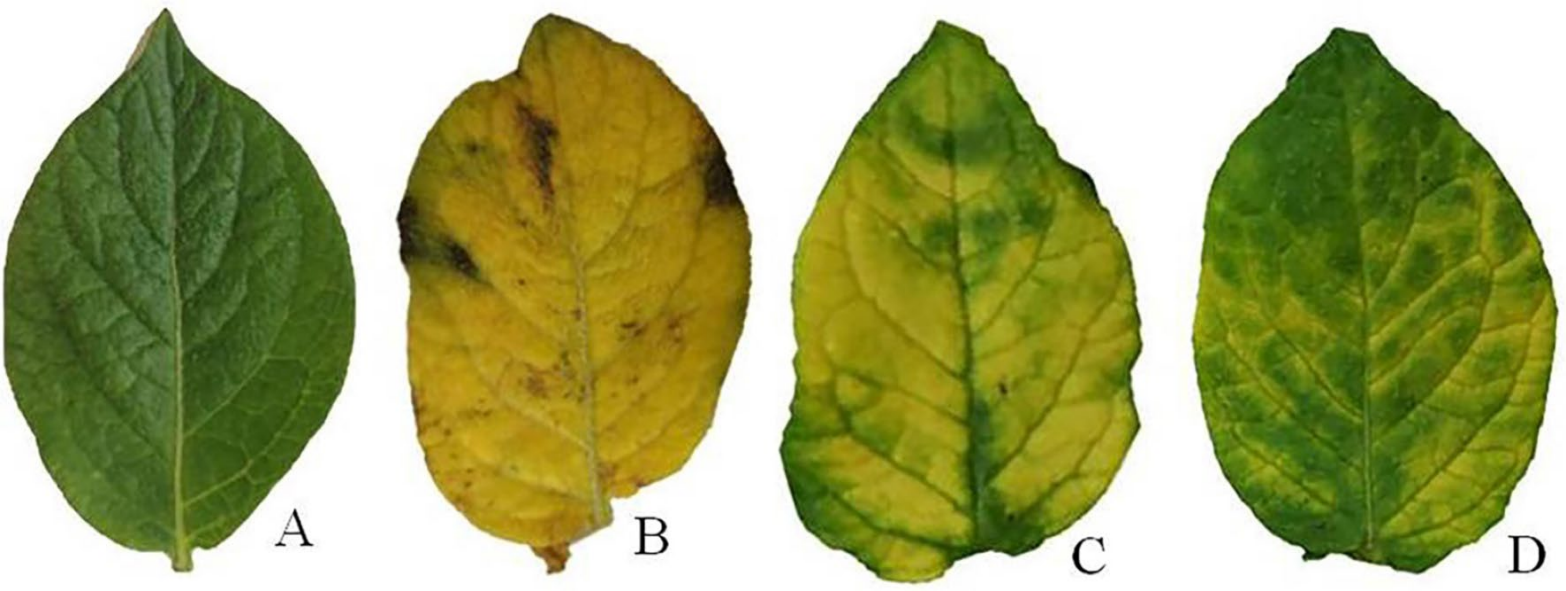
A photograph showing the disease symptoms on potato leaves infected with AMV at 50 days post inoculation. Where A: Mock-treated plants (T1), B: plants inoculated with AMV only (T2), C: plants treated with CF 24 h before inoculation of AMV (T3) and D: plant treated with CF, 24 h before inoculation of AMV and 24 h after inoculation with AMV (T4).

The symptoms of AMV including yellow blotching and bright mottling ended with clear visibility of calico symptoms on non-treated infected potato plant leaves (T2) were observed at 21 dpi (Fig. 1). The symptom appearance on POT1 treated plants 24 h before viral inoculation (T3) and POT1 treated plants before and after viral inoculation (T4) treatment was delayed approximately three and five days, respectively, compared to T2 treatment.

**Figure 2:**
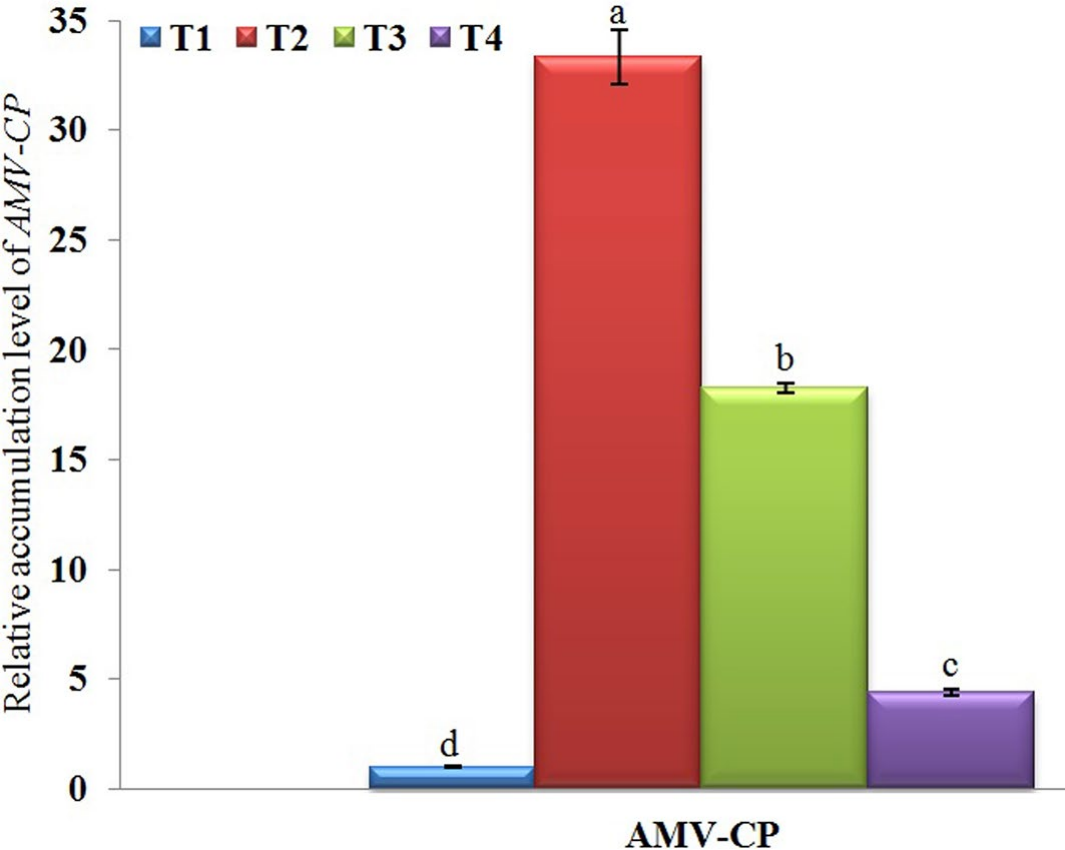
A histogram showing the relative expression level of *AMV-CP* gene in AMV-infected potato plants at 21 days post inoculation. Where, T1 = Mock-treated plants (Control), T2 = plants inoculated with AMV only, T3 = plants treated with CF 24 h before inoculation of AMV, and T4 = plant treated with CF, 24 h before inoculation of AMV and 24 h after inoculation with AMV. Columns represent mean value from three biological replicates and bars indicate Standard Deviation (± SD). Significant differences between samples were determined by one-way ANOVA using CoStat software. Means were separated by Least Significant Difference (LSD) test at *P* ≤ 0.05 levels and indicated by small letters. Columns with the same letter means do not differ significantly.

Comparing to T1 plants, T2 plants exhibited the higher accumulation level of AMV (33.33-fold) in AMV-infected potato leaves. On the other hand, a significant decrease in the viral accumulation level in T3 (18.23-fold) and T4 (4.40-fold) treatment plants was observed (Fig. 2).

The data of the potato plant parameters from the greenhouse experiment showed significant reduction in tuber number, tuber weight, fresh weight and dry weight of potato plants that were infected with AMV (T2), recording 2.33 ± 0.58 g, 22.32 ± 1.09 g, 20.12 ± 1.07 g, and 2.46 ± 0.07 g, respectively, compared to control plants (Table 2). The foliar applications of POT1 either T3 or T4 were significantly increased potato tuber numbers, tuber weights, fresh weights and dry weights in comparison to AMV-infected potato plants (T2). The T4 treatment accomplishes the adverse effects of the disease by increasing tuber number or weight with 4.33 ± 0.58 and 60.26 ± 2.23 g, respectively. Moreover, the fresh weight as well as the dry weight of the T4 treatment were significant greater than those of the other treatments at 70 dpi. No disease symptoms were observed on the non-infected plants.

**Table 2:** Effect of foliar applications *Bacillus licheniformis* culture filtrate on the growth parameters of potato plants infected with AMV at 70 days post inoculation. T1 = Mock-treated plants (Control), T2 = plants inoculated with AMV only, T3 = plants treated with CF 24 h before inoculation of AMV, and T4 = plant treated with CF, 24 h before inoculation of AMV and 24 h after inoculation with AMV. *Values of each column followed by the same letter are not significantly different according to the least significant differences (LSD) test (*P* ≤ 0.05), each value represents the mean of five replicates ± SD.

Treatments *	Tuber number	Tuber weight (g)	Fresh weight (g)	Dry weight (g)
T1	4.33 ± 0.58^a^	61.81 ± 1.08^a^	26.03 ± 0.69^b^	2.74 ± 0.07^b^
T2	2.33 ± 0.58^c^	22.32 ± 1.09^c^	20.12 ± 1.07^d^	2.46 ± 0.07^c^
T3	4.00 ± 1.00^b^	50.13 ± 1.00^b^	23.12 ± 1.37^c^	2.67 ± 0.37^b^
T4	4.33 ± 0.58^a^	60.26 ± 2.23^a^	32.32 ± 1.00^a^	3.62 ± 0.22^a^

### Transcriptional levels of polyphenol biosynthetic pathways-related genes

It is well known that, plant polyphenolic compounds, secondary metabolites, play important roles in plant growth and defense against different biotic and abiotic stresses. The transcriptional expression levels of thirteen genes encoding the essential enzymes regulating the polyphenol biosynthetic pathways were investigated at 21 dpi. The three-phenylpropanoid, chlorogenic and flavonoid biosynthetic pathways are the major route of polyphenol biosynthetic compounds.

### The core phenylpropanoid biosynthetic pathway

**Figure 3:**
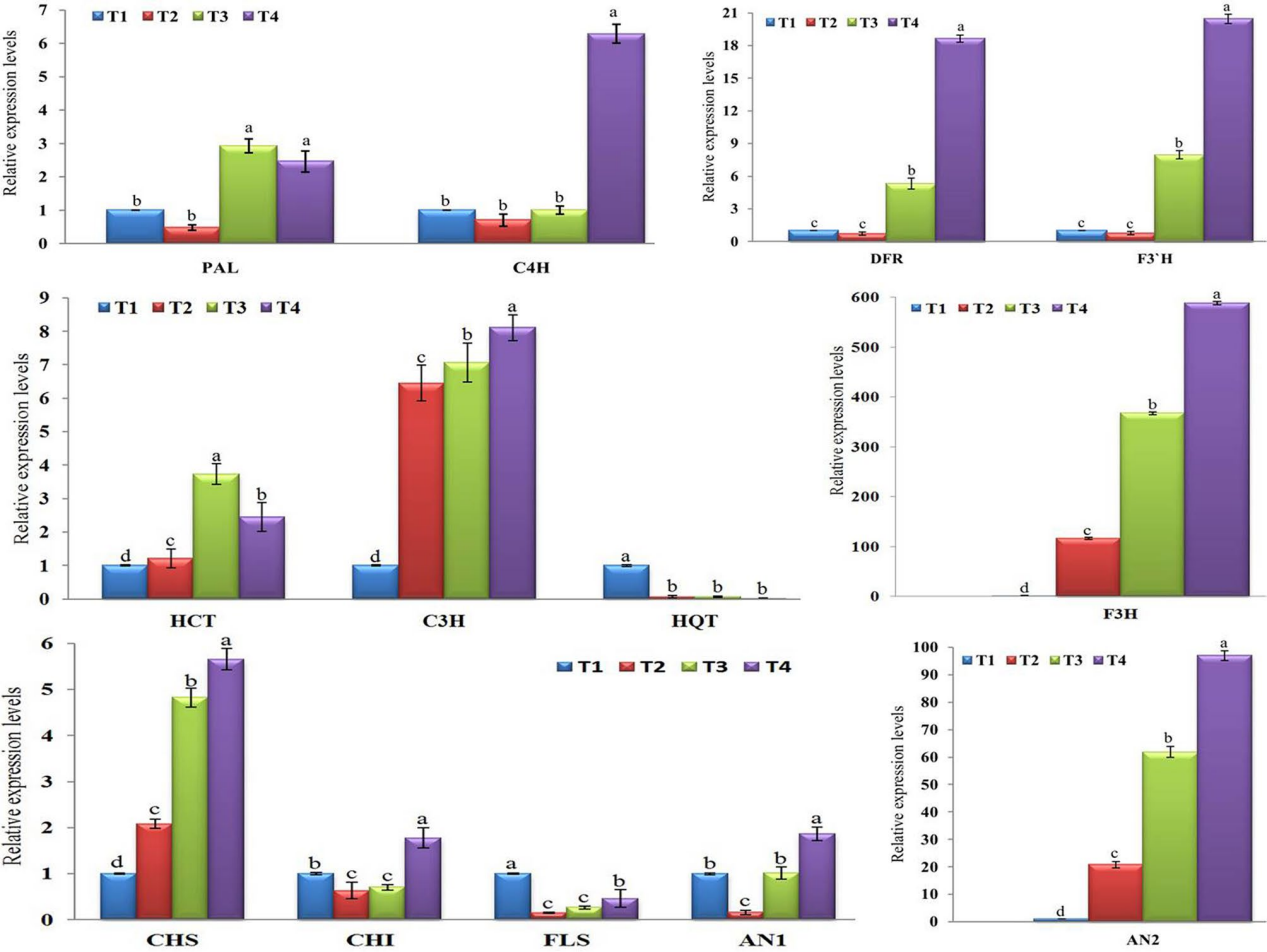
A histogram showing the relative transcriptional expression levels of polyphenol (phenylpropanoid, chlorogenic acid, and flavonoid) biosynthetic pathways genes in AMV-inoculated potato plants at 21 days post inoculation. Where, T1 = Mock-treated plants (Control), T2 = plants inoculated with AMV only, T3 = plants treated with CF 24 h before inoculation of AMV, and T4 = plant treated with CF, 24 h before inoculation of AMV and 24 h after inoculation with AMV. Columns represent mean value from three biological replicates and bars indicate Standard Deviation (± SD). Significant differences between samples were determined by one-way ANOVA using CoStat software. Means were separated by Least Significant Difference (LSD) test at *P* ≤ 0.05 levels and indicated by small letters. Columns with the same letter means do not differ significantly.

The relative expression levels of the two genes, *PAL* and *C4H*, encoding the first two enzymes in phenylpropanoid biosynthetic pathway were evaluated. Compared to control (T1), a significant up-regulation of *PAL* with a relative expression of 2.928- and 2.462-fold change, no significant changes, was observed in T3 and T4 treatments, respectively (Fig. 3). Despite *PAL* exhibited down-regulation with relative expression levels of 0.479-fold in T2 treatment, there was no significant change with control (Fig. 3). On the other hand, *C4H* was induced and significantly up-regulated in T4 treatment plants with transcript level 6.291-fold increased than control (Fig. 3). Like *PAL*, *C4H* was also down-regulated in T2 treatment with a relative expression level of 0.703-fold, while it was quite equal in T3 treatment compared with the control plants, there was no significant change between T1, T2 and T3 (Fig. 3). Consequently, foliar spraying of potato plants with CF 24 h before inoculation with AMV (T3) induced expression of *PAL* only, while the dual treatments (T4), foliar spraying of potato plants with CF 24 h before inoculation with AMV and 24 h after inoculation, triggered the expression of both *PAL* and *C4H*, genes.

### The chlorogenic acid biosynthetic pathway

The transcript levels of three genes (*HCT*, *C3H* and *HQT*) encoding three regulatory enzymes of chlorogenic acid biosynthesis were investigated. The significant up-regulation of *HCT* was observed in all treatments with relative expression levels of 1.203-, 3.732- and 2.441-fold change for T2, T3 and T4, respectively, compared to control (Fig. 3). The highest transcript level (8.111-fold) of *C3H* was observed in T4 treatment, followed by T3 and T2 with a relative transcript level of 7.06- and 6.453-fold change, respectively, compared to control (Fig. 3). These results showed that AMV infection induced expression of *HCT* and *C3H* in potato and the expression increased with POT1 treatments either before or after infection. On the other hand, the obtained data indicated that the *HQT* gene was not induced neither AMV infection nor POT1 treatments. The down-regulation with relative expression levels of 0.062-, 0.070- and 0.018- for T2, T3 and T4, respectively, was observed (Fig. 3).

### The flavonoid biosynthetic pathway

Compared to control, a clear differentiation in transcriptional profiles of eight genes (*CHS*, *CHI*, *F3H*, *FLS*, *DFR*, *F3′H*, *AN1*, and *AN2*) encoding eight enzymes controlling the flavonoid biosynthesis pathway was observed (Fig. 3). For *CHS* expression, up-regulation with a significant relative expression level (2.084-fold) was observed in AMV-infected plants (T2) when compared with the control plants. However, POT1-treated plants before infection (T3) or before + after (T4) exhibited higher expression levels with 4.823- and 5.656-fold change, respectively (Fig. 3). Thus, the treatment with either AMV or POT1 can trigger *CHS* expression level. Concerning *CHI*, the dual CF treatment (T4) showed the best results of *CHI* gene expression. The expression level was induced only in T4 treat-plants with a significant relative expression level of 1.777-fold-chang, while T2 and T3 showed down-regulation with relative expression levels of 0.636-and 0.703-fold, respectively, lower than control (Fig. 3). Among tested genes, *F3H* was the highest induced gene in all treatments when compared with control (Fig. 3). However, AMV-infected plant (T2) showed a high expression level (116.162-fold), the POT1-treated plants before 24 h of AMV inoculation (T3) exhibiting higher transcriptional level (367.092-fold). Moreover, the stronger transcript level of *F3H* (588.133-fold) in T4 treated-plants reflects the higher inducing activity of both AMV and POT1 for *F3H* transcription. Unexpectedly, the transcript level of *FLS* gene was shutdown in all treatments in comparison with control (Fig. 3). The down-regulation with relative expression levels of 0.147-, 0.266- and 0.463-fold in T2, T3 and T4, respectively, compared with control were observed. It was noted that the expressions of *DFR* and *F3′H* were very similar to each other. Although T2 treated plants exhibited a reduction of *DFR* and *F3′H* by relative expression levels 0.712- and 0.750- fold, respectively, no significant changes were reported when compared with control (Fig. 3). On the one hand, T3 treated plants showed up-regulation with significant transcript levels 5.314- and 7.963-fold change for *DFR* and *F3′H*, respectively. Additionally the dual POT1 treatment (T4) was more enhancer than T3, resulting in the highest expression levels of 18.635- and 20.440-fold increased than control for *DFR* and *F3′H*, respectively (Fig. 3). Concerning *AN1* gene, significant up- and down-regulations with relative expression levels of 1.866- and 0.162-fold change were showed in T4 and T2 treated-plants when compared with control (Fig. 3). For *AN2* transcript level, significant up-regulation in the transcription levels in all treatments was observed when compared with control. The highest induction with a relative expression level (97.005-fold) was showed in T4 treatment, while T2 treated-plants exhibited expression level of 20.821-fold change than control (Fig. 3).

### Phytochemical constituents of the potato leaf extract

**Figure 4:**
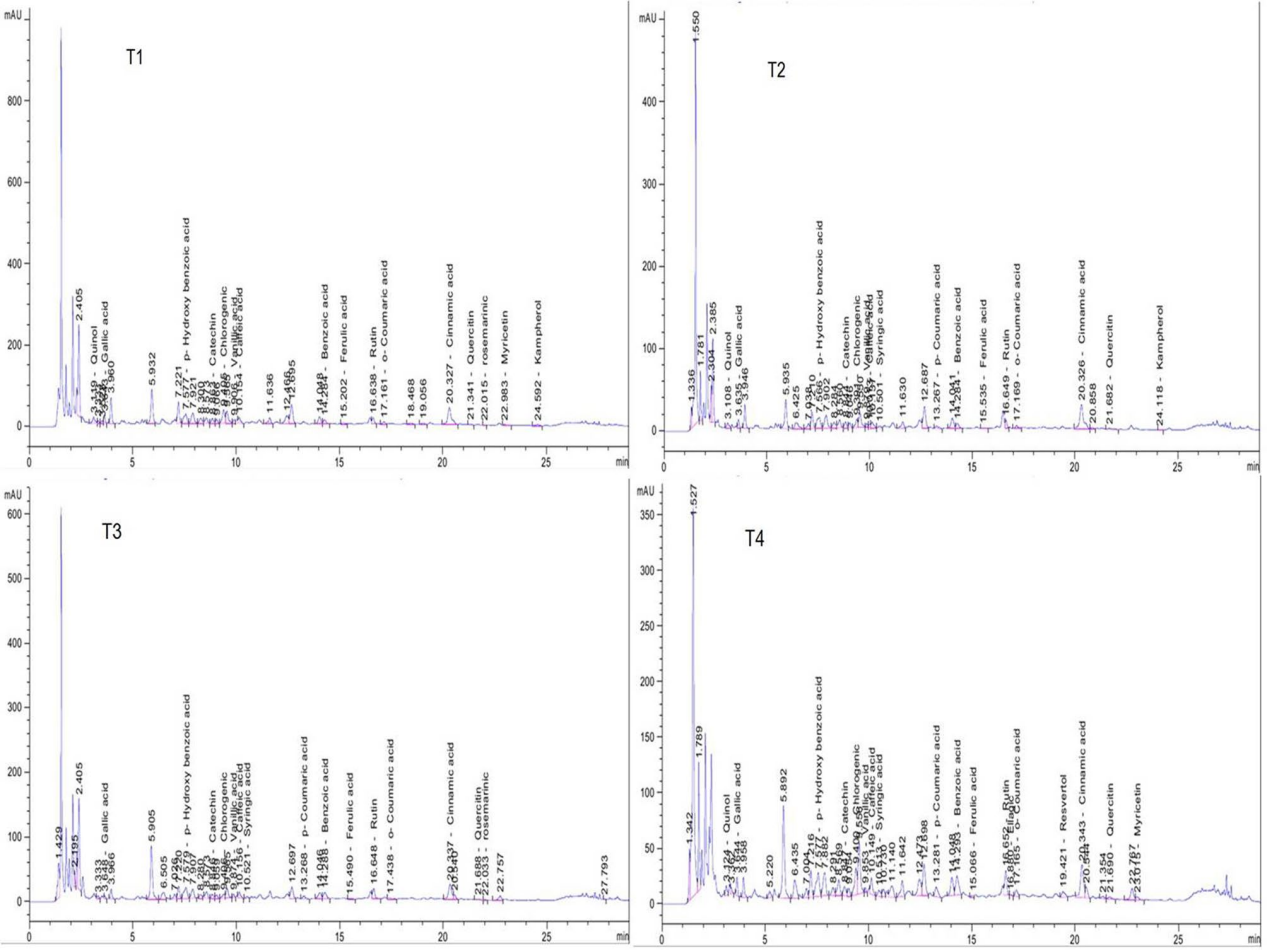
HPLC chromatograms of polyphenolic compounds identified in ethanol extract of potato leaves at 21 days post inoculation of different treatments. Where, T1 = Mock-treated plants (Control), T2 = plants inoculated with AMV only, T3 = plants treated with CF 24 h before inoculation of AMV, and T4 = plant treated with CF, 24 h before inoculation of AMV and 24 h after inoculation with AMV.

The HPLC chromatograms of ethanolic extracts of T1, T2, T3 and T4 potato plants were shown in Fig. 4. HPLC analysis revealed that the total contents of 20 polyphenolic compounds were 7,534.91, 1606.49, 1621.55, 7,218.86 mg/kg for T1, T2, T3 and T4, respectively, (Table 3). The main identified phenolic compounds were benzoic acid, *p*-Hydroxy benzoic acid, chlorogenic acid, vanillic acid, caffeic acid, syringic acid, *p*-Coumaric acid, ferulic acid, resvertol and cinnamic acid while flavonoid compounds were catechin, rutin, myricetin, quercetin and kaempferol. The most abundant phenolic compounds (mg/kg) were benzoic acid (2,420.31, 598.22, 717.70 and 3,166.29), *p*-Hydroxy benzoic acid (1,152.13, 338.95, 295.39 and 860.58), chlorogenic acid (445.84, 55.62, 29.29 and 241.58), cinnamic acid (418.52, 123.54, 58.04 and 180.31) in T1, T2, T3 and T4 extracts, respectively.

**Table 3:** HPLC chemical composition analysis of polyphenolic compounds in ethanol potato leaves extracts. Where, T1 = Mock-treated plants (Control), T2 = plants inoculated with AMV only, T3 = plants treated with CF 24 h before inoculation of AMV, and T4 = plant treated with CF, 24 h before inoculation of AMV and 24 h after inoculation with AMV.

Compound	Concentration (mg/kg)			
	T1	T2	T3	T4
Benzoic acid	2,420.31	598.22	717.70	3,166.29
*p*-Hydroxy benzoic acid	1,152.13	338.95	295.39	860.58
Chlorogenic acid	445.84	55.62	29.29	241.58
Cinnamic acid	418.52	123.54	58.04	180.31
Vanillic acid	114.69	18.78	15.87	25.44
Caffeic acid	112.01	9.83	21.67	78.27
*o*-Coumaric acid	45.48	15.11	9.48	34.21
Catechin	70.38	15.89	10.80	40.12
Ferulic acid	38.49	2.61	3.24	11.63
Rutin	812.98	170.40	331.25	992.24
Quercetin	232.10	91.81	58.03	281.80
Rosemarinic	131.28	–	40.02	–
Myricetin	428.79	–	–	438.40
Quinol	629.14	–	–	–
Kaempferol	340.30	19.97	–	–
Gallic acid	–	–	11.42	–
Ellagic acid	–	–	–	4.42
Resvertol	–	–	–	495.51
Syringic acid	–	11.69	10.03	53.93
*p*-Coumaric acid	–	7.65	9.32	52.99
Total	7,534.91	1606.49	1621.55	7,218.86

**Figure 5:**
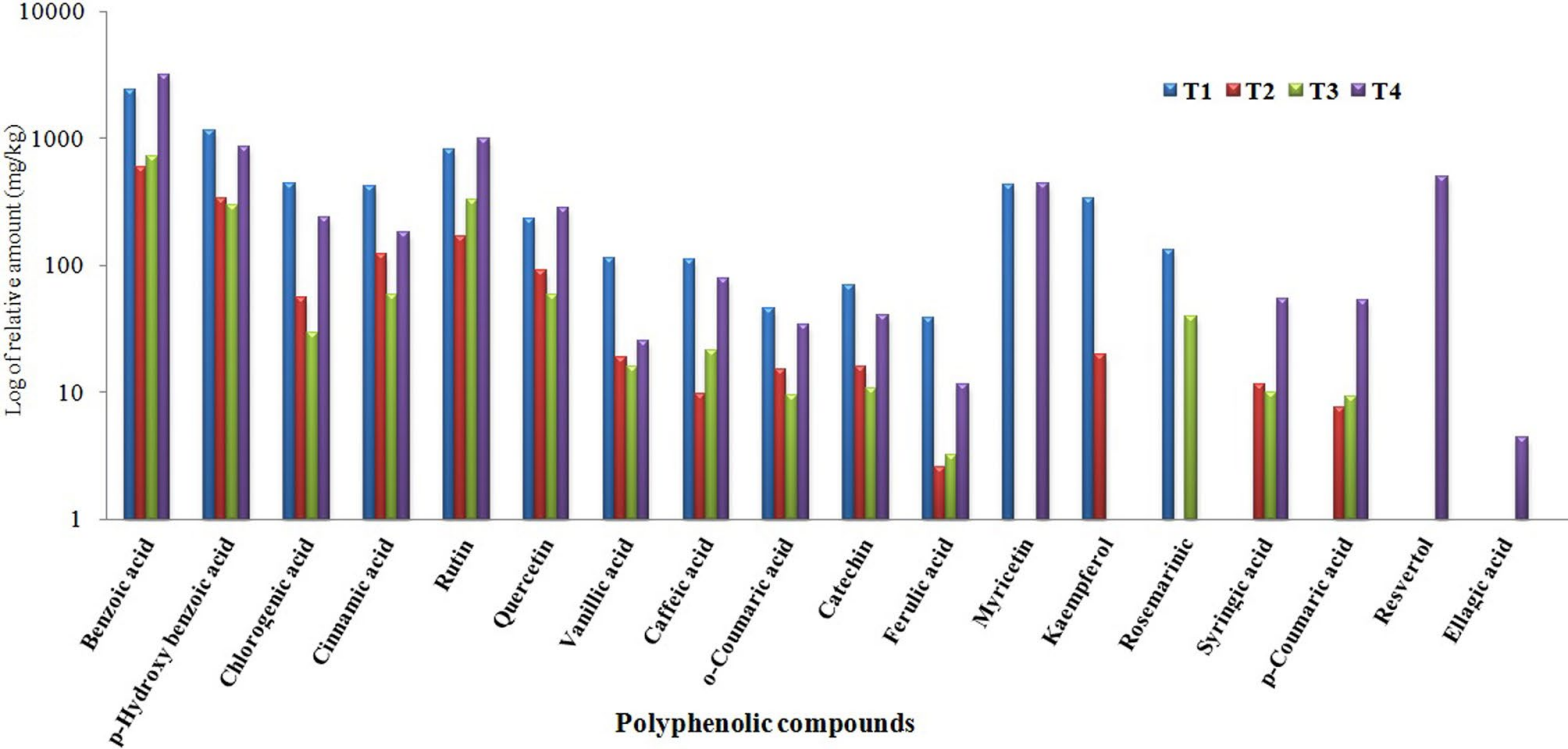
A histogram showing comparison of log of relative accumulation levels of polyphenolic compounds identified in ethanol extract of potato leaves at 21 days post inoculation of different treatments. Where, T1 = Mock-treated plants (Control), T2 = plants inoculated with AMV only, T3 = plants treated with CF 24 h before inoculation of AMV, and T4 = plant treated with CF, 24 h before inoculation of AMV and 24 h after inoculation with AMV.

Compared to control (T1), syringic acid and *p*-Coumaric acid were induced only in T2, T3 and T4 with accumulation levels of (11.69, 10.03 and 53.93 mg/kg), (7.65, 9.32 and 52.99 mg/kg), respectively, while Ellagic acid and resvertol were induced in T4 only with 4.42 and 495.51 mg/kg, respectively, (Fig. 5). On the other hand, the three flavonoid compounds (Rutin, Myricetin and Quercetin) were overexpressed in T4 plants with accumulation content (992.24, 438.40 and 281.80 mg/kg, respectively) when compared to T1 plants (812.98, 428.79 and 232.10 mg/kg, respectively).

### Identification of bioactive metabolites

**Figure 6:**
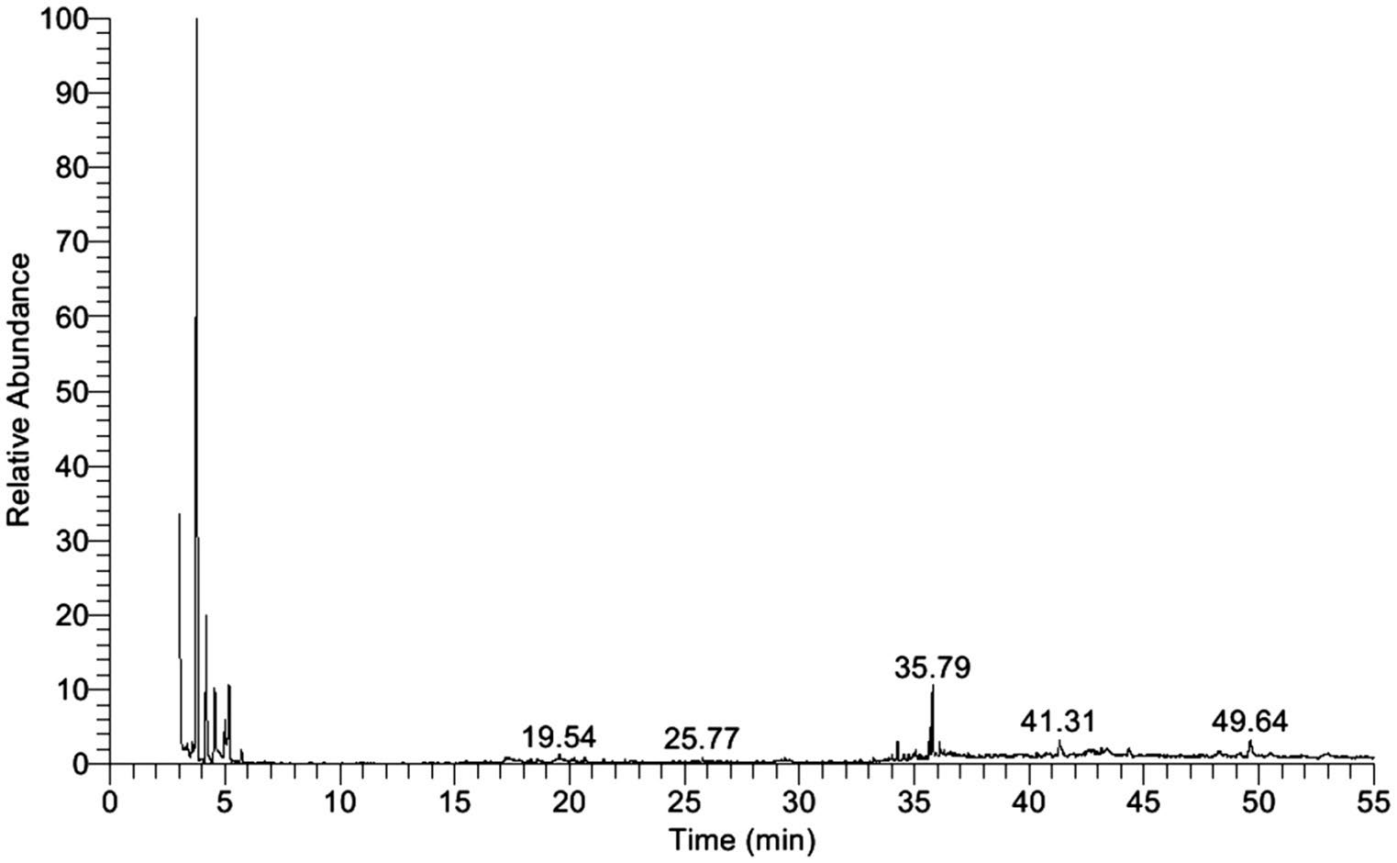
Histogram showing Gas chromatography-mass spectrometry (GC-MS) fractionation of ethyl acetate extract of *Bacillus licheniformis* strain POT1.

The identification of the bioactive components of bacterial filtrate extract was done by the GC-MS machine. The active constituents with their retention time (RT), detected compounds, chemical formula (CF), and chemical structures were illustrated in Table 4. The GC-MS analysis of the ethyl acetate filtrate extract (Fig. 6) showed 22 bioactive compounds with a high concentration of Pyrrolo[1,2-a]pyrazine-1,4-dione that having retention time 35.79 and the highest peak area, followed by 2,5-Piperazinedione,3,6-bis(2-methylpropyl)- having retention time 41.31 (RT) and Pyrrolo[1,2-a]pyrazine-1,4-dione, hexahydro-3-(phenylmethyl) having RT 49.64. Other constituents ranged with varying retention time and peak area.

**Table 4:** The chemical properties of the five major compounds of ethyl acetate extract of *Bacillus licheniformis* culture filtrate using GC-MS analysis.

Retention time (min)	Detected compounds	Chemical formula	Molecular weight (g/mol)	Molecular structure
19.54	Benzenepropanoic acid, dodecyl ester	C_21_H_34_O_2_	318	
25.77	Heptadecane,2,6,10,15-tetramethyl-	C_21_H_44_	296	
35.79	Pyrrolo[1,2-a]pyrazine-1,4-dione	C_11_H_18_N_2_O_2_	210	
41.31	2,5-Piperazinedione,3, 6-bis(2-methylpropyl)-	C_12_H_22_N_2_O_2_	226	
49.47	Pyrrolo[1,2a]pyrazine1,4-dione, hexahydro-3-(phenylmethyl)-	C_14_H_16_N_2_O_2_	244

## Discussion

Plant viruses are among the most important plant pathogens, as near half of the emerging epidemics have a viral etiology, causing problems of food security and they are responsible for huge losses of crop production^29,30^. Due to negative impacts on public health and environmental hazards, chemical treatments including pesticide or insecticide must be managed and controlled. Biological control using PGPR (one or more strains) is being considered as an alternative or a supplemental way of handling of plant diseases better than the chemical control in agriculture^31–34^. Consequently, searching and discovering new environmental eco-friendly biocontrol agents capable to control plant viral diseases are demand^8^. In the current study, the antiviral activity of *Bacillus licheniformis* strain POT1 against AMV on potato (*Solanum tuberosum* L.) plant was evaluated. Moreover, the transcriptional levels of thirteen genes involved in phenylpropanoid, chlorogenic acid and flavonoid biosynthetic pathways genes, as well as bioactive constituents of POT1 crude filtrate (CF), were analyzed. Additionally, HPLC analysis was used to compare the content of polyphenol compounds with the expression levels of biosynthetic pathway genes. To our knowledge, this is the first time to deal with the effect of PGPR against AMV in potato plants.

Under greenhouse experiments, the application of POT1 has significantly increased plant growth, yield, reduced disease severity, and virus accumulation compared to infected potato plants without any treatments. We have shown that inhibition of AMV infection can be generated by treating plants with bacterial culture filtrate either prior to or post-challenge with the virus. Our results showed that the up-regulation of almost genes triggered by bacterial filtrate resulted in increasing resistance to AMV via limiting viral accumulation, symptom severity, and growth parameters increasing. Many reports showed that the application of some *Bacillus* spp., *Pseudomonas* spp. and *Streptomyces* spp., improved plant growth and increased protection against viral infection^35–38^.

In the current study, the obtained results showed that potato plants infected with AMV (T2) were significantly reduced the tuber numbers and weight as well as fresh and dry weight when compared with the control plants (T1). However, T4 treatment was significantly enhanced and improved all evaluated growth parameters recording the highest values of fresh and dry weight when compared to the other treatments and significantly reduced the negative effects resulted from the viral infection.

The greenhouse experiment results confirmed the effective biocontrol activity of POT1 against AMV infection, which resulted in a considerable decrease in AMV concentration levels. Significant reductions in virus concentration by 45.29% and 86.79% for potato leaves of T3 and T4 treatments, respectively, were showed when compared to T2 treatment. These results suggest that CF of POT1 contains secondary metabolites that can play a notable role in SAR. In this context, the foliar application of *Streptomyces* spp. CF exhibited a significant reduction of PVY in potato^39^. Thus, POT1 activates induced systemic resistance (ISR) of potato plants against AMV infection. ISR using PGPR showed promising results against plant viruses such as tomato mottle virus, CMV, and PVY in tomato^40^ cucumber^41–43^ and potato^44,45^, respectively. Besides the activation of some defense genes, ISR enhanced the production peroxidase, antioxidant protective enzyme and secondary metabolites^46,47^. Among the secondary metabolites, polyphenolic compounds play vital roles in plant growth and resistance against different biotic and abiotic stresses^19^. Routes to the major classes of polyphenol compounds involve three pathways (i) phenylpropanoid pathway, (ii) chlorogenic acid pathway and (iii) flavonoid pathway^27^.

The phenylpropanoid pathway started with the conversion of L-phenylalanine by *PAL* to cinnamic acid then to *p*-coumaric acid by cinnamic acid 4-hydroxylase (*C4H*) and ended by the formation of the main intermediate coumaroyl-CoA that is controlled by 4-coumarate-CoA ligase (4CL). Based on the correlation between accumulation rates and resistance degree, phenylpropanoid compounds have been proposed to play crucial roles in plant defense against different microbial pathogens^48–50^. In the current study, the expression of *PAL* was induced in T3 and T4 treatments, while T2 treatment showed down-regulation. On the other, *C4H* was induced and overexpressed with a relative expression level 6.291-fold change in treatment T4 only. Consistent with the results of the transcriptional levels, HPLC analysis revealed that *p*-coumaric acid was induced and accumulated with the highest amount (52.99 mg/kg) in T4 treatment. Thus, overexpression of *C4H* could be associated with *p*-coumaric acid content increasing. The down-regulation of both genes, *PAL and C4H*, in infected potato tissues (T2) reflects the suppressor activity of AMV. Many researchers reported that the viral infection was associated with down-regulation or decreasing in *PAL* and *C4H* activity^25,51,52^. Besides its role in plant defense, *PAL* involved in the biosynthesis of salicylic acid (SA) that plays an essential role in the induction of plant systemic resistance pathogens^50,53–55^. Moreover, through enhancing the accumulation of lignin in the plant cell walls, *C4H* plays a role in increasing plant defense^56,57^. Consequently, the induction of *PAL* and *C4H* transcripts in T3 and T4 treatment suggesting that POT1 is a good elicitor activated ISR that is associated with SA and secondary metabolites, precursors of chlorogenic acid and flavonoids, biosynthesis in potato tissues.

Chlorogenic acid, which is the ester of caffeic acid and quinic acid, is one of the polyphenol compounds, phenolic acids, that improving plant disease resistance through inhibiting pathogens^58,59^**.** The chlorogenic acid pathway started with the conversion of *p*-coumaroyl CoA to shikimate through *HCT* catalyzing activity^60^. Following synthesis of *p*-coumaroyl shikimate by *C3H*, *HCT* catalyzes the transfer of it to the caffeoyl CoA^61^ ended with chlorogenic acid through *HQT* activity^62^. In the present study, although *HCT* showed slightly induced after AMV infection in T2 treatment (1.203-fold), the overexpression with relative expression level 3.732- and 2.441-fold change was observed in T3 and T4 treatments, respectively. Likewise, the up-regulation of *C3H* with the transcriptional expression level of 6.453-, 7.061- and 8.111-fold was reported in T2, T3 and T4, respectively. *HCT* and *C3H* involved in lignin biosynthesis in the plant cell wall^56,63^. Thus, the induction of transcriptional expression of these genes shows their protective role against AMV and suggests that the potato plant can use the lignifications as one of its defense to resist the viral infection and movement. On the other hand, the decreasing of chlorogenic and caffeic acids contents upon AMV-inoculation (T1, T2 and T3) could reflect the down-regulation of transcriptional expression levels of *HQT* for these treatments when compared to control. Tomato *HQT* overexpression was associated with increases in chlorogenic acid content and versa versa^62^. Based on current results, whether *HQT* suppression and *HCT* and *C3H* induction, AMV-infected potato plant was associated with decreasing the content of chlorogenic acid. We can assume that AMV could not make complete suppression of chlorogenic acid biosynthesis and had a suppression effect on *HQT* rather than *HCT* and *C3H*. Moreover, the ISR activated by POT1 may be correlated with inducing and increasing cell wall lignifications.

The flavonoid pathway started with the conversion of *p*-coumaroyl CoA to naringenin chalcones, through *CHS* catalyzing activity, which can be transformed to naringenin by the action of *CHI*^27^. These are the first two steps of the flavonoid pathway and are strictly required for chalcones and dihydrochalcones production, which considered being the primary precursors and constituting the main intermediates for a large number of flavonoids synthesis by the action of enzymes set such as *F3H*, *FLS*, *DFR*, *F3′H*, *AN1* and *AN2*^64,65^. Although AMV induced *CHS* (2.084-fold) in infected potato tissues (T2), increasing transcriptional expression levels in T3 and T4 by 4.823- and 5.656-fold change, respectively, were observed. On the other hand, T4 treatment showed a significant up-regulation of *CHI* with expression level 1.777-fold increased than control. The repression of *CHI* in T2 and T3 suggested that AMV infection suppressed naringenin biosynthesis, even if the potato plants treated with CF 24 h before viral infection. Interestingly the dual foliar application (T4), before and after the viral infection, showed the highest induction of both genes, *CHS* and *CHI*, that are strictly required for flavonoid production in multiple tissues of potato^27,65^.

The obtained results showed that *F3H*, the key enzyme in flavonoid biosynthesis in plants^66^, was the master expressed gene among flavonoid pathway genes with the highest expressions level 116.162-, 367.092- and 588.133- fold change than the control for T2, T3 and T4, respectively. Likewise, *F3′H* exhibited significant up-regulation in both POT1 treatments with relative expression 7.963- and 20.440-fold increased than control. *F3H* converts hydroxylate naringenin to dihydroflavonol or dihydrokaempferol while *F3′H*, the primary enzymes responsible for the diversification of anthocyanins, transforms dihydrokaempferol into dihydroquercetin^67,68^. The stronger induction of *F3H* and *F3′H*, suggesting that they played significant roles in plant defense against viral infection and leads to induction of biosynthesis of many intermediate precursors compounds for flavonoids production. The six significant subclasses of flavonoids are the flavones, isoflavones, flavonols, flavanones, flavan-3-ols and anthocyanidins. The high expression of *F3H* was associated with the accumulation of both quinochalcones and flavonols in safflower plants^69^. Among flavonols^18^, myricetin and quercetin were up-regulated and accumulated in T4 plants with 438.40 and 281.80 (mg/kg), respectively. The antiphytoviral activity of quercetin against TMV^70^, PVX^71^ and Tomato ringspot virus^72^ were previously reported. Meanwhile, POT1 T4-treatment was associated with induction and accumulations of many flavonoids compounds.

Due to their powerful antioxidant properties, anthocyanins (flavonoids) protect plants against various biotic and abiotic stresses^73–75^. *DFR*, a NADPH-dependent reducing enzyme, converts dihydroflavonols to leucoanthocyanidins which necessary for formation of anthocyanins in higher plants^76^. Quattrocchio et al.^77^ reported that anthocyanin pathway is regulated at the *DFR* step in Petunia hybrid plants. Comparing to control in this study, both T3 and T4 treatments induced *DFR* transcripts with relative expression values of 5.314- and 18.635-fold change, respectively. Based on this data, we are suggesting that POT1 treatments stimulated the plant immune defense system to produce more anthocyanins-related compounds. *AN1* and *AN2* are two transcription factors involved in the regulation of anthocyanin biosynthesis^78^. Thus, a significant up-regulation (1.866-fold) of *AN1* in T4 treatment could be induced biosynthesis of anthocyanin-related compounds. D’Amelia et al.^79^ reported that *AN1* expression is associated with high anthocyanin contents in leaves. On the other hand, the higher and stronger expression of *AN2* with relative expression levels of 61.819- and 97.005-fold change in T3 and T4, respectively, revealed that anthocyanins played important roles in plant defense against viral infections. HPLC analysis showed that, a significant overaccumulation of the total phenolic contents in T4 (7,218.86 mg/kg) rather than T2 (1606.49 mg/kg) plants. Meanwhile, D’Amelia et al.^1^ showed that *AN2* able to regulate the production of phenolic compounds and high expression in potato tuber during drought stress was associated with increases in total phenolic levels^27^. The accumulation of anthocyanin in plants upon biotic stresses has been reported. *Ustilago maydis* triggered anthocyanin induction in maize^80^, and anthocyanin-enriched tomato fruits exhibited lower susceptibility to gray mold^81^. Moreover, antibacterial, antifungal and antiviral activities of certain anthocyanins were also reported^82–84^.

Bacillus species produce wide structural variability of secondary metabolites that exhibiting strong antibacterial and antifungal activities^85,86^. Moreover, it represents a new and rich source of secondary metabolites that need to be discovered. The GC-MS spectrum analysis showed that pyrrolo[1,2-a] pyrazine-1,4-dione was the major compound in POT1 ethyl acetate extract. Pyrrole was known for a wide range of bioactivities, including antibiotics, antitumor, antifungal, anti-inflammatory, anti-angiogenesis and cholesterol reducing drugs^87^. Moreover, pyrrolo[1,2-*a*]pyrazine-1,4-dione isolated from *Streptomyces* spp. showed antioxidant^88^ and anticandidal^89^ activity, while that isolated from *Shewanella* spp. exhibited anticyanobacterial and algicidal activity^90^. Additionally, these compounds showed excellent protease inhibitor activity with a very good antiretroviral activity^91^ and have the ability to inhibit HIV-1 viruses, DNA polymerases and protein kinase activity^92,93^. The obtained results supported previous reports of pyrrolo[1,2-a] pyrazine-1,4-dione activity in preventing viral replication. Consequently, POT1 could be useful as a preventive agent against AMV infection. However, further examinations needed for the potential field application.

## Material and methods

### Plant materials

Virus-free potato tubers cv Spunta used in this study were kindly provided by the International Potato Center (CIP), Ministry of Agriculture and Land Reclamation, Egypt.

### Source of the viral isolate

Alfalfa mosaic virus Kh1 isolate (Acc# MN099289) used in this study was previously isolated from infected potato plants^52^ and maintained continuously on *Nicotiana glutinosa* plants under greenhouse conditions.

### Source of bacterial isolate, biochemical tests characterization and culture filtrate preparation

Soil-adhered potato roots were collected from a potato field in Alexandria governorate, Egypt. The roots were crushed in a mortar and a loopful was cultured on Nutrient Agar (NA) media and incubated at 30 °C. Different colonies were picked and assayed for antiviral activity using half-leaf method^94^. The bacterial isolate showing a maximum antiviral activity was selected, and preliminary identified based on morphological and biochemical characteristics^95^. For the bacterial culture filtrate (CF), the selected bacterial isolate was grown in a nutrient broth medium and incubated on a shaking incubator at 30 °C for two days. The bacterial culture was centrifuged at 6,000 rpm for 10 min at 4 °C to separating bacterial cells and collecting supernatants.

### DNA extraction, 16 rRNA amplification and sequencing analysis

Bacterial genomic DNA was isolated from bacterial culture of selected bacterial isolate using Wizard Genomic DNA Purification Kit (Promega, USA) according to the manufacture instructions. By using 16 rRNA specific primers, forward (5`-AGAGTTTGATCCTGGCTCAG-3`) and reverse (5`-GGTTACCTTGTTACGACTT-3`), PCR reaction was performed as previously reported^96^. Briefly, PCR reaction was started with initial denaturation at 95 °C for 2 min, followed by 35 cycles at 95 °C for 30 s, 50 °C for 30 s and 72 °C for 1.5 min. An additional final extension step was carried out at 72 °C for 5 min. PCR amplified products were checked on 1.5% agarose gel electrophoresis, visualized under UV transilluminator, and purified by a PCR clean-up column kit (QIAGEN, Germany) for sequencing. Sanger sequencing of 16 rRNA gene was performed using a BigDye Terminator v3.1 Cycle Sequencing kit and a 3130xl Genetic Analyzer system (Applied Biosystems, USA). After the sequencing process, the annotated nucleotide sequence was analyzed using NCBI-BLAST (https://blast.ncbi.nlm.nih.gov/Blast.cgi), and deposited in Genbank. The phylogenetic tree was constructed based on the UPGMA statistical method with a bootstrap of 2.000 replicates using the MEGA 5 software^97^.

### Greenhouse experimental design

Potato tubers were cultivated in plastic pots (30 cm in diameter) filled with 4 kg of sterilized soil compromised of clay: sand, equal ratio (1:1). The experiment was carried out in four treatments, each comprised five replicate pots, and one potato tuber/each pot. After 35 days of growing, two true upper leaves of each potato plants were dusted with carborundum and mechanically inoculated, as previously described^98^. The first treatment (T1) was mock-treated plants (control), in which potato plants inoculated with free-virus inoculation buffer + foliar spraying of free bacterial broth medium. The second treatment (T2) was plants inoculated with AMV with foliar spraying of free bacterial broth medium (infected). The third treatment (T3) included plants treated by foliar spraying of CF, 24 h before inoculation of AMV. The fourth treatment (T4) was potato plants treated by twice foliar spraying of CF, 24 h before inoculation of AMV and 24 h post-inoculation of AMV. All plants were kept under insect-proof greenhouse conditions 28 °C/16 °C (day/night) and 70% relative humidity and daily observed for symptoms development recording.

### Plant samples collection, total RNA extraction and cDNA synthesis

Leaves of five biological replicates of each treatment were collected at 21 dpi and kept at -80 °C until use. Total RNA was extracted using the RNeasy plant mini kit (QIAGEN, Germany) according to the manufacturer’s instructions. Each biological sample was a mix of five samples derived from five different plants. The extracted RNA was dissolved in DEPC-treated water, treated with RNase-free DNase to eliminate genomic DNA, quantified by NanoDrop UV spectrophotometer (Labtech International Ltd, Sussex, UK) and the integrity was assessed by agarose gel electrophoresis. Two micrograms of total RNA for each sample were reverse transcribed to cDNA using oligo (dT) and random hexamer primers with reverse transcriptase enzyme of Super-Script II (Invitrogen, USA), according to the manufacturer’s instructions. The reverse transcriptase reactions were performed in a thermal cycler (Eppendorf, Germany), according to Behiry et al.^99^. The amplified cDNA was used as a template for quantitative real-time PCR (qRT-PCR).

### Quantitative Real-Time PCR (qRT-PCR) assay and data analysis

Different primer sets specific for polyphenolic-related genes were synthesized according to previous studies (Table 5). The housekeeping gene *EF1-α* (Table 5) was used as a reference gene in order to normalization of the transcript expression levels^52^. Each sample in all reactions was run in triplicate using Rotor-Gene 6000 (QIAGEN, ABI System, USA) with the SYBR Green PCR Master Mix (Fermentas, USA) and performed according to previously reported^100^. The amplification program of the thermal cycler included an initial denaturation step at 95 °C for 10 min, followed by 40 cycles consisting of; denaturation at 95 °C for 15 s, annealing at 60 °C for 30 s and extension at 72 °C for 30 s. After that, the melting curves were obtained to eliminate the inclusion of non-specific products. The relative expression level of the target gene was accurately quantified and calculated according to 2^-ΔΔCT^ algorithm^101^.

**Table 5:** Nucleotide sequences of the real-time RT-PCR primers used in this study.

Primer name	Abbreviation	Direction	Sequence (5′–3′)	Amplicon length
Phenylalanine ammonia-lyase	*PAL*	Forward	ACGGGTTGCCATCTAATCTGACA	92
		Reverse	CGAGCAATAAGAAGCCATCGCAAT	
Cinnamic acid 4-hydroxylase	*C4H*	Forward	CCCAGTTTTTGGAAATTGGCTTCA	104
		Reverse	GCCCCATTCTAAGCAAGAGAACATC	
Hydroxycinnamoyl Co A shikimate hydroxycinnamoyl transferase	*HCT*	Forward	TCTCCAACCCCTTTTAACGAACC	103
		Reverse	CAACTTGTCCTTCTACCACAGGGAA	
p-coumarate 3-hydroxylase	*C3H*	Forward	TTGGTGGCTACGACATTCCTAAGG	100
		Reverse	GGTCTGAACTCCAATGGGTTATTCC	
Hydroxycinnamoyl Co A quinate hydroxycinnamoyl transferase	*HQT*	Forward	CCCAATGGCTGGAAGATTAGCTA	99
		Reverse	CATGAATCACTTTCAGCCTCAACAA	
Chalcone synthase	*CHS*	Forward	CACCGTGGAGGAGTATCGTAAGGC	93
		Reverse	TGATCAACACAGTTGGAAGGCG	
Chalcone isomerase	*CHI*	Forward	GGCAGGCCATTGAAAAGTTCC	103
		Reverse	CTAATCGTCAATGATCCAAGCGG	
Flavanone 3-hydroxylase	*F3H*	Forward	CCAAGGCATGTGTGGATATGGACC	103
		Reverse	CCTGGATCAGTATGTCGTTCAGCC	
Flavonol synthase	*FLS*	Forward	CCTCCTTCCTACAGGGAAGCAAA	91
		Reverse	CAAGCCCAAGTGACAAGCTCCTAA	
Dihydroflavonol 4-reductase	*DFR*	Forward	TCACAGGAGCAGCTGGATTTATCG	91
		Reverse	TCAGGATCACGAACAGTAGCATGG	
Flavonoid 3′ hydroxylase	*F3′H*	Forward	TGGGTATACCCAAACTCATTCCG	96
		Reverse	AAAAGCCCAAAGTTGATGTGAAAGG	
Anthocyanin 1 transcription factor	*AN1*	Forward	CCTCAACCTCAGAAATTCAGAAGC	102
		Reverse	TCGTTGTTGTTGTCGTTCGATGC	
Anthocyanin 2 transcription factor	*AN2*	Forward	ACAAGATGCCACTTTCCTTCACC	101
		Reverse	TGTGCATCGTTGGGAGTTAGG	
Elongation factor 1-alpha	*EF1-α*	Forward	ATTGGAAACGGATATGCTCCA	101
		Reverse	TCCTTACCTGAACGCCTGTCA	
Alfalfa mosaic virus-coat protein	*AMV-CP*	Forward	CCATCATGAGTTCTTCACAAAAG	151
		Reverse	TCGTCACGTCATCAGTGAGAC	

### Accumulation level of *AMV-CP*

By using a specific primer of *AMV-CP* (Table 5), qRT-PCR was performed using the SYBR Green PCR Master Mix (Fermentas, USA) to detect AMV and its level in the tested potato leaves. The reaction consisted of a 20 μL mixture containing 1 μL cDNA (50 ng), 1 μL of 10 pmol μL^−1^ of each primer (forward and reverse), 10 μL of 2 × SYBR Green PCR Master Mix and 7 μL of nuclease-free water. The qRT-PCR reaction was performed using a Rotor-Gene 6000 in two steps. The first step at 95 °C for 10 min as initial denaturation step and the second step cycle consisting of 40 cycles (95 °C for 15 s, 60 °C for 30 s and 72 °C for 30 s). Each biological sample was run in triplicate to guarantee data reproducibility. The relative accumulation level of *AMV-CP* was calculated as according to described above.

### Yield and growth parameters evaluation

In AMV infectivity experiments, plants from each treatment were carefully uprooted, washed under running water, and assessed for the fresh weight (g), dry weight (g), tuber weight (g) and tuber number/each plant. After drying plant samples in an oven at 80 °C for 72 h, dry weights were determined and calculated in all treatments (20 plants in total) at 70 dpi.

### Ethanol extract preparation and HPLC analysis conditions

Potato leaves of all treatments were collected, air-dried, and milled. About 2 g of the leaves were macerated and extracted with 15 mL of 96% ethanol for 5 h in a shaking water bath at 35 °C. After filtration and centrifugation, the cleared supernatant was transferred to another tube and concentrated by evaporation at a temperature below 35 °C. The ethanol extract was stored in a brown vial prior to HPLC analyses. An Agilent 1260 Infinity HPLC series, equipped with a Quaternary pump and a Zorbax Eclipse plus C18 column (100 mm × 4.6 mm i.d.) (Agilent Technologies, CA, USA), operated at 30 °C, was used to identify the phenolic and flavonoid-type compounds according to Al-Huqail et al.^102^. Separation was achieved using a mobile phase consisting of mixture of solvent A (HPLC grade water containing 0.2% phosphoric acid), solvent B (acetonitrile) and solvent C (methanol). The injection volume was 20 μL while VWD detector was set at 284 nm. The standard polyphenolic compounds used were Quinol, Gallic acid, Catechol, p-Hydroxy benzoic acid, Chlorogenic acid, Vanillic acid, Caffeic acid, Syringic acid, *p*-Coumaric acid, Benzoic acid, Ferulic acid, Ellagic acid, o-Coumaric acid, Resvertol, Cinnamic acid, Rosemarinic, Catechin, Rutin, Myricetin, Quercetin, Naringenin and Kaempferol.

### GC-MS fractionation of bacterial ethyl acetate extract

To identify active components of bacterial culture filtrate, a 48 h bacterial culture broth was precipitated, and the supernatant was collected and mixed with ethyl acetate, as a solvent, in the ratio of 1:1 (v/v). The mixture was shaken vigorously for 20 min, and by using separating funnel, the ethyl acetate phase was separated from the aqueous phase. Ethyl acetate extract was concentrated by evaporation at 50 °C in a rotary evaporator. The residue which contained the secondary metabolites and chemical compounds was analysed using gas chromatography-mass spectroscopy (GC-MS)^103^. The analyses were run on a GC-MS system (TRACE 1300 Series, Thermo, USA) and the test carried out at the Marine Pollution Lab of National Institute of Oceanography and Fisheries, Alexandria, Egypt. The mass detector used in split mode and helium gas with a flow rate of 1 ml/min was used as a carrier. The injector was operated at 250 °C and oven temperature for initial setup was at 60 °C for 2 min, scan time 0.2 s; mass range 50–650 amu and ramp 4/min to 250 °C for 20 min. Mass spectra were taken at 70 eV, during the running time 53 min. The constituents were identified after comparing them with available data in the GC-MS library in the literature.

### Statistical analysis

The relative expression values of three replicates for each set were analysed by one-way ANOVA using the CoStat software. The significant differences of the relative expression levels were determined according to the least significant differences (LSD) *P* ≤ 0.05 level of probability, and standard deviation (± SD) is shown as a column bar. Compared to mocked-inoculated potato tissues, the relative expression values higher than 1 was demonstrated an increase in the gene accumulation (up-regulation), while values lower than 1 means a decrease in expression levels (down-regulation).
